# Does Goal Conflict Necessarily Undermine Wellbeing? A Moderated Mediating Effect of Mixed Emotion and Construal Level

**DOI:** 10.3389/fpsyg.2021.653512

**Published:** 2021-06-02

**Authors:** Wujun Sun, Zeqing Zheng, Yuan Jiang, Li Tian, Ping Fang

**Affiliations:** ^1^Faculty of Education, Henan Normal University, Xinxiang, China; ^2^Beijing Key Laboratory of Learning and Cognition, Department of Psychology, Capital Normal University, Beijing, China; ^3^Department of Psychology, Beijing Sport University, Beijing, China

**Keywords:** goal conflict, life satisfaction, mixed emotions, construal level, moderated mediating effect

## Abstract

Development occurs through the process of setting and working toward goals, in which individuals are often working toward multiple goals that are likely to conflict with one another. Although motivation theories hold that goal conflict is a kind of potential stress that may damage individuals’ mental health and wellbeing, the empirical research results on the relationship between goal conflict and wellbeing are quite different. There may be unknown factors affecting the relationship between the two. Against this background, we conducted the exploration of the relationship between goal conflict and life satisfaction, mainly by analyzing the moderated mediating effect of mixed emotions and construal level. The results showed that the goal conflict did not directly affect life satisfaction (*β* = −0.01, *p* > 0.5) but indirectly influenced life satisfaction through mixed emotions (*β* = −0.17, *p* < 0.001). The construal level moderated the relationship between mixed emotions and life satisfaction (*β* = −0.08, *p* < 0.01), and the higher construal level will predict higher life satisfaction especially when mixed emotions were low (*M* − *SD*) or medium (*M*). Therefore, the hypothesis of moderated mediating effect is verified, and we can draw the following conclusions: (1) Goal conflict does not necessarily impair life satisfaction. (2) Goal conflict impairs life satisfaction conditional on the fact that it triggers mixed emotions. Since mixed emotions are often accompanied by feelings of ambivalence and discomfort, they reduce the individual’s evaluation of life satisfaction. (3) In the path of goal conflict reducing life satisfaction through mixed emotions, the higher construal level mitigates the adverse effects of mixed emotions to some extent.

## Introduction

The development of mature individuals is achieved through the process of setting and working toward goals (Deci and Ryan, 2008; Hooker, 2015). As the core motivational structure affecting behavior, goals can enrich individuals psychologically by providing them with meaning for their activities and selves (West et al., 2013). Research evidence shows that the pursuit of meaningful goals is associated with healthy psychological function and individual performance (Covington, 2000; Locke and Latham, 2002; Freund and Hennecke, 2015).

However, most people have often more than one ideal goal at some point in their lives. The multi-goal pursuit and goal conflict among personal life are deemed to a part of daily life (Riediger and Freund, 2008; Unsworth et al., 2014). For example, students in a university usually strive to do well in their classes. Meanwhile, they socialize with others to make new friends. Sometimes, they also want to improve their abilities by taking a job in a student union or doing a part-time job to strive for economic independence. However, due to limited time and energy, these goals cannot be achieved at the same time. When the pursuit of one valuable goal hinders the pursuit of another or when the plans and behaviors of two or more goals are incompatible, goal conflict occurs (Austin and Vancouver, 1996; Boudreaux and Ozer, 2013).

How conflicting goals affect wellbeing? The answer seems obvious. Early psychologists, such as Hull (1938) and Maslow (1943), recognized the negative psychological significance of conflict or goal conflict in their theories, and modern motivation theories also regard goal conflict as a potential source of psychological stress (McNaughton and Gray, 2000; DeYoung, 2015). Unfortunately, in empirical research, the relationship between goal conflict and wellbeing is not very clear, and the relevant research results showed a large divergence (Gray et al., 2017). Most studies have found a significant positive correlation between goal conflict and lower life satisfaction, more depression, and anxiety symptoms (Emmons, 1986; Kelly et al., 2011; Boudreaux and Ozer, 2013); however, some other study results showed there was no significant correlation between goal conflict and subjective wellbeing (King et al., 1998; Romero et al., 2009). In a few studies, researchers even found that lower-level goal conflict is related to goal importance, anticipated happiness on goal success, and positive affect (Freitas et al., 2009). It is undeniable that these contradictory research results may be partly caused by research methods, measurement tools, and sample characteristics (Kelly et al., 2015; Gray et al., 2017). But then, it may also be that there are still unfound mediators and moderators between goal conflict and subjective wellbeing, which make the relationship between them and show the variable results in different situations or conditions. For example, it was found that conflicts of daily activities in adolescents and students were associated with health symptoms, but not in adults (Perring et al., 1988), which may be due to differences in cognitive characteristics and emotional responses between adolescents and adults. Therefore, the research on the mediating and moderating factors of goal conflict affecting wellbeing will help to reconcile the inconsistency and divergence in the empirical research results, and present more details of the relationship between goal conflict and wellbeing, and improve the understanding of the mechanism of these two. Life satisfaction, as an important part of wellbeing and a key parameter for measuring the quality of life, is widely used in the research on the relationship between goal conflict and wellbeing (Kelly et al., 2015), so this study also used life satisfaction as an indicator of wellbeing to specifically explore the mediating and moderating effects of emotional and cognitive factors between goal conflict and life satisfaction.

Emotion connects the life experience with goals and actions and is an indispensable element in exploring the relationship between goal conflict and happiness. Studies have shown that in the process of multi-goal pursuit and goal conflict, individuals usually experience mixed emotions rather than pure positive or negative emotions (Berrios et al., 2017; Mejía and Hooker, 2017). According to the evaluative space model, positive and negative emotions are two independent variables, and there are three activation modes, such as independent activation, co-inhibition, and co-activation (Cacioppo and Berntson, 1994). Mixed emotional experience is the product of the co-activation of the positive and negative emotional systems, which is manifested in the complex emotional states that both positive and negative emotions experience simultaneously (Larsen et al., 2001; Larsen, 2017; Burkitt et al., 2019). As individuals working toward a higher level of goal or toward multiple goals, the importance and position of these goals in the target level may be similar, such as work and family. People are not sure which goal should take priority. The achievement of one goal can impede the progress of others. The gains come with the losses. All of these situations contain both positive valence information and negative valence information, which provides the conditions for the generation of mixed emotions (Shuman et al., 2013; Berrios et al., 2015a).

In addition, the research on the functions of mixed emotions shows that, although in the long run, the ability to react to difficult moments in life can be seen as an effective way to regulate pain, foster mental resilience, and maintain physical and mental health (Hershfield et al., 2013; Braniecka et al., 2014); however, in terms of short-term influence, the mixed emotional state characterized by the co-occurrence of opposite valence emotions can cause contradictory feelings and discomfort to different degrees, which will lead to negative evaluation and negative decision making (Hong and Lee, 2010; Penz and Hogg, 2011; Bee and Madrigal, 2013). Life satisfaction is an immediate evaluation of the current life, which is more likely to be influenced by the emotional state at that time. Moreover, the mixed emotions induced by goal conflict should be a highly simultaneous mode in which both positive and negative emotions increase simultaneously (Oceja and Carrera, 2009; Barford et al., 2020), which is inconsistent with the individual’s needs for decision making and behavior in this context, and needs to be resolved immediately. Therefore, it is often more contradictory, confusing, and uncomfortable, thus reducing the individual’s evaluation of life satisfaction. From the above research results, we seem to be able to infer that when goal conflict occurs, the mixed emotions experienced by individuals increase, which is accompanied by a strong sense of contradictory feelings and discomfort, resulting in individuals’ lower evaluation of life satisfaction. Based on the previous research, Hypothesis 1 is as follows:

Hypothesis 1: Mixed emotions play a mediating role in the impact of goal conflict on life satisfaction.

In the context of goal conflict, the emotional experience of opposite valence tends to make individuals trapped in contradictory psychological states and then feel pain and discomfort. A high construal level and future-oriented thinking may help to deal with this situation (Mejía and Hooker, 2017). Construal level denotes the different abstract levels of the representation of objects, and this abstract level is of a different degree (Trope and Liberman, 2003). The high construal level reflects a generalized understanding of behaviors or events and is superordinate and out of context. The low construal level reflects the specific detail or circumstance of the behaviors or events, which are subordinate and context dependent. Studies have shown that individuals with high construal levels have less discomfort and a more positive attitude due to mixed emotions (Hong and Lee, 2010). Compared with individuals with low construal levels, individuals with high construal levels may have a better ability to experience and cope with mixed emotions (Sun et al., 2021). The reasons may involve several aspects: Individuals with high construal level are more tolerant to mixed emotions and are more likely to adopt flexible and creative processing methods (Förster et al., 2004). They are more willing to choose desirable but demanding actions (Carrera et al., 2020) and have better self-control (Fernández et al., 2018). The increase in psychological distance induced by high construal level can also alleviate the intensity and vividness of mixed emotions experienced by people (Wong and Bagozzi, 2005). Thus it can be seen that, in the face of mixed emotions caused by goal conflict, construal level can not only affect attitudes to mixed emotions and reactions, but also adjust the intensity of the emotional response, to a certain extent which determines whether individuals will experience a strong sense of discomfort and produce negative evaluation when facing goal conflict and mixed emotions. Based on this background information, Hypothesis 2 is as follows:

Hypothesis 2: The mediating effect of mixed emotions between goal conflict and life satisfaction will be moderated by construal level.

Hypothesis 2a: The moderating effect may appear in the first and second half of the mediating pathway, and higher construal level will predict lower mixed emotions and higher life satisfaction.

Hypothesis 2b: The moderating effect may appear in the first half of the mediating pathway, and higher construal level will predict lower mixed emotions.

Hypothesis 2c: The moderating effect may appear in the second half of the mediating pathway, and higher construal level will predict higher life satisfaction.

**Figure 1:**
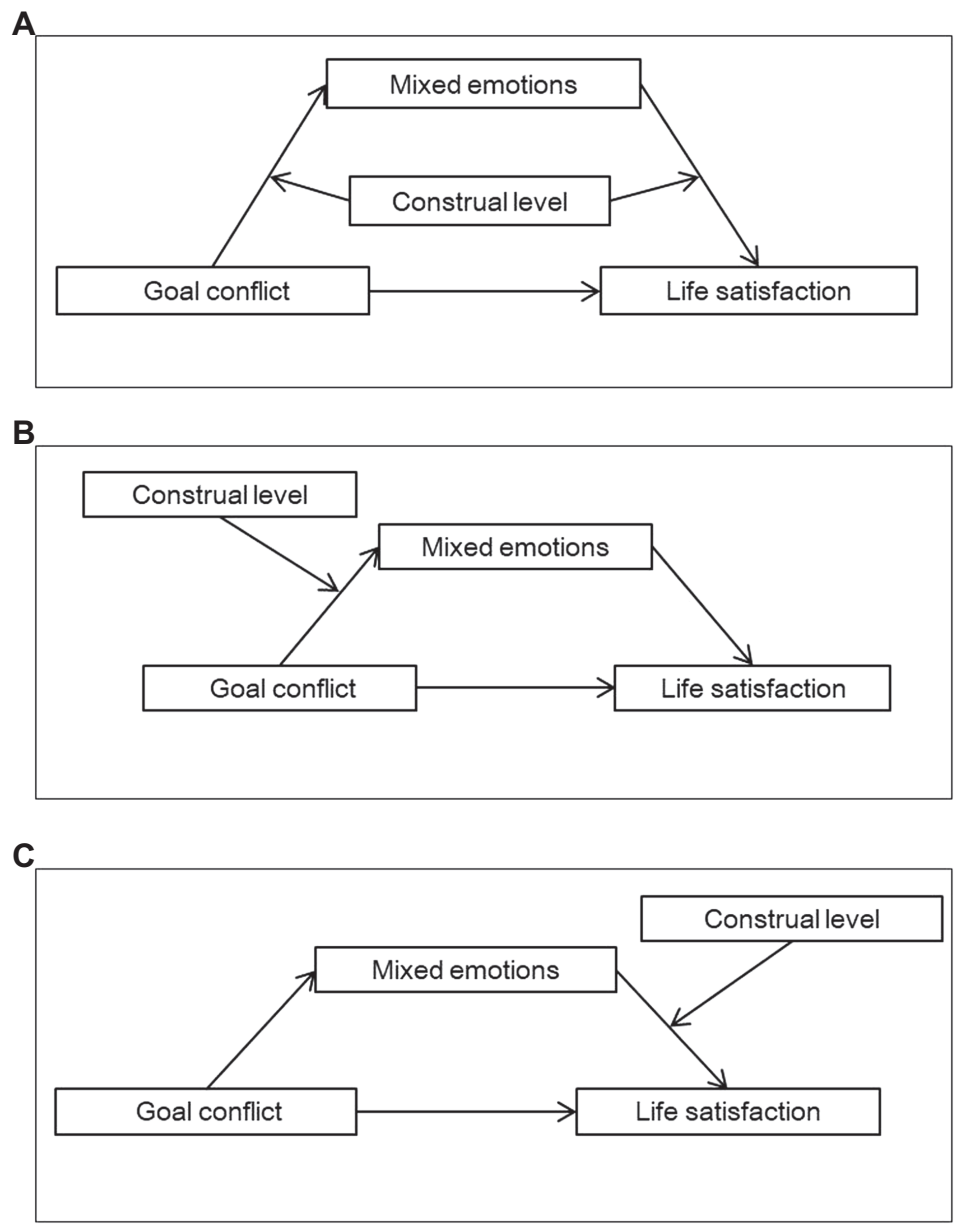
The moderated mediating models.

In summary, this study constructed a moderated mediating model to test whether mixed emotions mediate the relationship between goal conflict and life satisfaction and whether construal level can moderate this mediation process. Hypothesis 2a-2c’s theoretical models are shown in Figures 1A-C respectively.

## Materials And Methods

### Participants

Considering that the age period between 18 and 24 is the peak of life meaning seeking (Bodner et al., 2014), which is a critical period for comparison and choice of various life goals, and goal conflicts frequently occur, 846 college students from 19 provinces in China were selected as the research subjects in this age period. Participants included 487 male students (57.6%) and 359 female students (42.4%), whose average age was 19.73 years, *SD* = 1.51. There were 245 freshmen (29%), 291 sophomores (34.4%), 186 juniors (22%), and 124 seniors (14.7%). In terms of the major in university, there were 408 who majored in liberal arts (48.2%) and 438 who majored in science (51.8%).

The study used the Wenjuanxing platform^1^ to recruit participants and collect data. The respondents were informed about the volunteer and confidential nature of the study, and they provided their electronic informed consents prior to complete the online questionnaires. It took about 10 min to complete all the questionnaires.

### Measurements

#### Conflicting Goals Scale

Conflicting Goals Scale adapted by Berrios et al. (2018) based on the Strivings Instrumentality Matrix (Emmons and King, 1988) was used in this study. In this scale, participants need to answer three different questions according to the five most important goals of themselves, in order to assess the conflict degree of these goals over the last few days. For example, “I think that pursuing some of these goals hurts the pursuit of the other ones.” All items were rated on a 5-point Likert scale ranging from 1 (strongly disagree) to 5 (strongly agree), with higher scores indicating higher goal conflict level. In the previous study, the Cronbach’s *α* coefficient of the scale was 0.69 (Berrios et al., 2018), while in this study, the coefficient of the scale was 0.75, indicating fair internal consistency.

#### Satisfaction With Life Scale

The Satisfaction With Life Scale developed by Diener et al. (1985) was used to evaluate the subjects’ overall life satisfaction. The scale consists of five items, such as “I am satisfied with my life.” A 7-point Likert scale was used to rate all five items from 1 (strongly disagree) to 7 (strongly agree), with higher scores indicating higher life satisfaction. This scale is one of the most widely used tools to measure life satisfaction, with the Cronbach’s *α* coefficient which was between 0.80 and 0.89 (Pavot and Diener, 1993). In this study, the *α* was 0.89, with good internal consistency.

#### Mixed Emotions Scale

The Mixed Emotions Scale developed by Berrios et al. (2015a) was adopted in this study. There are four items on the scale to measure participants’ mixed emotional experience in recent days. For example, “I’m feeling a mixture of emotions.” A 5-point Likert scale was used to rate all items from 1 (none at all) to 7 (very strong), with higher scores indicating higher mixed emotions level. This scale is a common method to measure mixed emotions, which has been proved to have good reliability and validity (Berrios et al., 2015b), with the Cronbach’s *α* coefficient which was greater than 0.85 (Berrios et al., 2015a, 2018). In this study, *α* = 0.90, showing excellent internal consistency.

#### Behavior Identification Form

The Behavior Identification Form (BIF) developed by Vallacher and Wegner (1989) was used to evaluate the construal level of the participants. Based on the Chinese cultural background, we selected 12 items from the original scale (the similar practice be adopted in the study of Fujita et al., 2006), with each item representing a behavior, and each behavior was described in abstract and concrete ways. The score was 0 when the subjects chose the concrete description of the project, and 1 when they chose the abstract description. Finally, the scores of 12 projects were summed to obtain the total BIF score. A higher score indicates a higher construal level. In the original scale, the item-total correlations ranged from 0.28 to 0.48, and the internal consistency was 0.85 (Vallacher and Wegner, 1989). In this study, the reliability estimation method of binary variables provided by Raykov et al. (2010) was used to obtain the reliability coefficient of the BIF of 0.70, which met the standards of psychometrics.

### Data Analysis

Harman’s single-factor test was used to test the common method biases of all of the items in the four scales. The SPSS 22.0 statistical software was used to conduct descriptive statistics and the Pearson bivariate correlation analysis. According to the test method proposed by Hayes (2015), we used PROCESS macro 2.16 for SPSS to test moderated mediating effect models proposed in Hypothesis 1 and Hypothesis 2. To be conservative, gender and grade were included as control variables in the above analyses to exclude their influences. The percentile bootstrap method based on deviation correction was applied in the PROCESS macro program, and the robust standard errors and bootstrap confidence intervals of parameter estimation were obtained by sampling 5,000 bootstrap samples (each sample size was 846). Finally, the simple slope test was used to determine how the construal level moderates the relationship between goal conflict and mixed emotions or between mixed emotions and life satisfaction.

## Results

### Common Method Biases

The results of Harman’s single-factor test showed that six factors with eigenvalues greater than 1 were extracted. The variance explained by the maximum common factor, respectively, was 18.32% before rotation and 14.79% after rotation, which were all less than the 40% required by the critical standard (Podsakoff et al., 2003). Therefore, the influence of common method bias in this study is very low.

### Descriptive Statistics and Correlational Analysis

Both mixed emotions and life satisfaction were significantly different by grade (*F* = 3.12, *p* < 0.05, and partial $\;\eta^{2}\;$= 0.01; *F* = 3.81, *p* = 0.01, and partial $\;\eta^{2}$ = 0.01), and gender differences were also found in both construal level and life satisfaction (*t* = 4.37, *p* < 0.001, and *d* = 0.30; *t* = 2.60, *p* < 0.01, and *d* = 0.18). With gender and grade controlled, the descriptive statistics and correlation analysis results of all variables showed that life satisfaction was significantly negatively correlated with goal conflict and mixed emotions, and positively correlated with construal level. Construal level had a significant negative correlation with goal conflict and mixed emotions. Mixed emotions were significantly positively correlated with goal conflict (see Table 1).

**Table 1:** Descriptive statistics and correlation matrix of all variables.

		*M*	*SD*	1	2	3	4
1	Goal conflict	2.96	0.86	1			
2	Mixed emotions	2.49	0.92	0.38^***^	1		
3	Construal level	6.70	2.31	−0.10^**^	−0.14^***^	1	
4	Life satisfaction	4.15	1.35	−0.07^*^	−0.18^***^	0.12^**^	1

*N* = 846:

* *p* < 0.05,

** *p* < 0.01,

*** *p* < 0.001.

### Mediating Model Analyses

**Figure 2:**
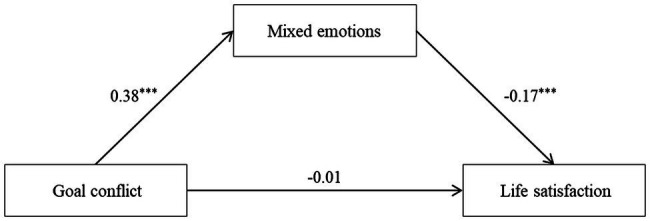
Path coefficients of goal confliction, mixed emotions, and life satisfaction. ^***^p < 0.001.

Correlation analysis results showed that the relationship between goal conflict, mixed emotions, and life satisfaction satisfied the condition for the mediating effect test. The Model 4 of PROCESS macro program for SPSS was used to test Hypothesis 1. The results in Table 2 show that goal conflict significantly positively predicted mixed emotions (*t* = 11.96, *p* < 0.001). When goal conflict and mixed emotions all entered the regression equation, mixed emotions significantly negatively predicted life satisfaction (*t* = −4.70, *p* < 0.001). The goal conflict had negative association with life satisfaction, but not significantly (*t* = −0.17, *p* > 0.5). The 95% bootstrap confidence interval of the mediation effect did not contain zero (−0.10, −0.04), and the indirect effect accounted for 91.17% of the total effect. Thus, mixed emotions played a mediating role in the relationship between goal conflict and life satisfaction, and Hypothesis 1 was supported. The detailed path model is shown in Figure 2.

**Table 2:** Regression analysis results of the mediating role of mixed emotions between goal confliction and life satisfaction.

Regression equation		Overall model fit			Significance of regression coefficient			
Outcome	Predictor	*R*	*R*^2^	*F*	*β*	LLCI	ULCI	*t*
Mixed emotions	Grade^a^	0.39	0.16	31.03^***^	−0.21	−0.37	−0.06	−2.66^**^
	Grade^b^				−0.14	−0.31	0.04	−1.52
	Grade^c^				−0.29	−0.49	−0.08	−2.79^**^
	Gender^d^				−0.04	−0.16	0.09	−0.58
	Goal conflict				0.38	0.32	0.44	11.96^***^
Life satisfaction	Grade^a^	0.22	0.05	7.35^***^	−0.06	−0.23	0.11	−0.71
	Grade^b^				−0.28	−0.47	−0.09	−2.95^**^
	Grade^c^				−0.02	−0.23	0.20	−0.17
	Gender^d^				0.15	0.02	0.29	2.20^*^
	Goal conflict				−0.01	−0.08	0.07	−0.17
	Mixed emotion				−0.17	−0.24	−0.10	−4.70^***^

Grade is dummy variable, Grade^a^: freshman = 0, sophomore = 1, junior = 0, and senior = 0; Grade^b^: freshman = 0, sophomore = 0, junior = 1, and senior = 0; Grade^c^: freshman = 0, sophomore = 0, junior = 0, and senior = 1; Gender^d^ is dummy variable, female = 0 and male = 1. Other variables in the model were normalized and then substituted into the regression equation. LLIC, lower-level confidence interval; ULIC, upper-level confidence interval.

* *p* < 0.05,

** *p* < 0.01,

*** *p* < 0.001.

### Moderated Mediating Model Analyses

The Model 21, Model 7, and Model 14 of PROCESS macro program for SPSS were used to test Hypotheses 2a, 2b, and 2c respectively. Of the three hypotheses, only Hypothesis 2c has been verified. Table 3 shows that in the direct path, goal conflict had a positive association with life satisfaction, but not significant (*β* = 0.01, *t* = 0.20, and *p* > 0.5). In the first half of the mediation path, goal conflict significantly positively predicts mixed emotions (*β* = 0.38, *t* = 11.96, and *p* < 0.001). In the second half of the mediation path, mixed emotions significantly negatively predicted life satisfaction (*β* = −0.17, *t* = −4.61, and *p* < 0.001), construal level significantly positively predicted life satisfaction (*β* = 0.10, *t* = 2.81, and *p* < 0.01), and the interaction term of construal level and mixed emotions significantly negatively predicted life satisfaction (*β* = −0.08, *t* = −2.61, and *p* < 0.01). The 95% bootstrap confidence interval for moderated mediating effects did not contain zero (−0.063, −0.003). When the construal level was divided into three levels according to the criteria of mean minus one standard deviation, mean, and mean plus one standard deviation, the 95% bootstrap confidence intervals for mediation effect of mixed emotions between goal conflict and life satisfaction were (−0.07, 0.01), (−0.10, −0.03), and (−0.15, −0.05). The results suggested that construal level had moderated the mediating effect of mixed emotions between goal conflict and life satisfaction, and Hypothesis 2c was supported.

**Table 3:** Regression analysis results of construal level moderate the mediation process.

Regression equation		Overall model fit			Significance of regression coefficient			
Outcome	Predictor	*R*	*R*^2^	*F*	*β*	LLCI	ULCI	*t*
Mixed emotions	Grade^a^	0.39	0.16	31.03^***^	−0.21	−0.37	−0.06	−2.66^**^
	Grade^b^				−0.14	−0.31	0.04	−1.52
	Grade^c^				−0.29	−0.49	−0.08	−2.79^**^
	Gender^d^				−0.04	−0.16	0.09	−0.58
	Goal confliction				0.38	0.32	0.44	11.96^***^
Life satisfaction	Grade^a^	0.26	0.07	7.48^***^	−0.06	−0.23	0.11	−0.72
	Grade^b^				−0.29	−0.47	−0.10	−3.05^**^
	Grade^c^				−0.02	−0.23	0.20	−0.16
	Gender^d^				0.13	−0.01	0.26	1.83
	Goal confliction				0.01	−0.06	0.08	0.20
	Mixed emotion				−0.17	−0.24	−0.10	−4.61^***^
	Construal level				0.10	0.03	0.16	2.81^**^
	Mixed emotion × Construal level				−0.08	−0.15	−0.02	−2.61^**^

Grade is dummy variable, Grade^a^: freshman = 0, sophomore = 1, junior = 0, and senior = 0; Grade^b^: freshman = 0, sophomore = 0, junior = 1, and senior = 0; Grade^c^: freshman = 0, sophomore = 0, junior = 0, and senior = 1; Gender^d^ is dummy variable, female = 0 and male = 1. Other variables in the model were normalized and then substituted into the regression equation. LLIC, lower-level confidence interval; ULIC, upper-level confidence interval.

** *p* < 0.01,

*** *p* < 0.001.

**Figure 3:**
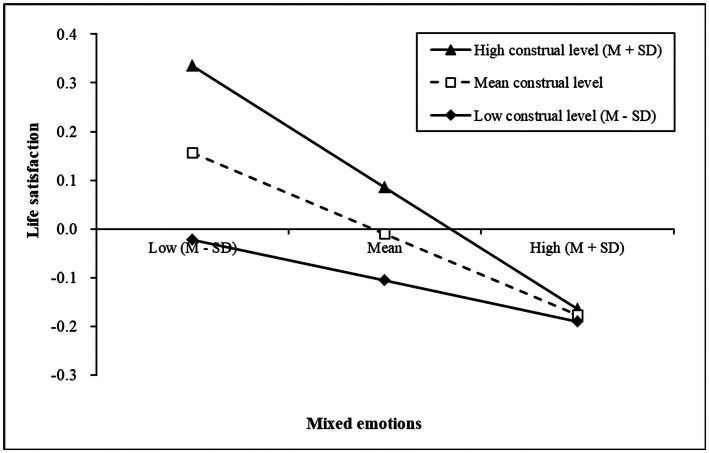
Simple slopes of construal level moderate the relationship between mixed emotions and life satisfaction.

Furthermore, a simple slope test was used to analyze the moderating effect of construal level between mixed emotions and life satisfaction, which is in the second half part of the mediation path. The results are shown in Figure 3. When construal level was high (*M* + *SD*) or medium (*M*), mixed emotions had a significant negative predictive effect on life satisfaction (*simple slope* = −0.25, *t* = −5.22, and *p* < 0.001; *simple slope* = −0.17, *t* = −4.90, and *p* < 0.001). When the construal level was low (*M* − *SD*), the negative predictive effect of mixed emotions on life satisfaction was not significant (*simple slope* = −0.08, *t* = −1.85, and *p* = 0.06). When mixed emotions were low (*M* − *SD*) or medium (*M*), individuals with high construal levels reported more life satisfaction than those with low construal level. However, when mixed emotions were high (*M* + *SD*), the reports of life satisfaction had no significant difference between individuals with high and low construal levels.

In summary, the moderated mediating model is established (Hayes, 2015). Mixed emotion mediated the relationship between goal conflict and life satisfaction, and the construal level moderated the mediating process of goal conflict affecting life satisfaction through mixed emotion.

## Discussion

Development occurs through the process of setting and working toward goals, in which individuals are often working toward multiple goals that are likely to conflict with one another. It is important for individuals to represent and solve goal conflict in an appropriate way that is related to personal achievement and healthy psychological function. The results of this study showed that goal conflict was negatively correlated with life satisfaction, which is consistent with most previous research results (Emmons, 1986; Boudreaux and Ozer, 2013; Kelly et al., 2015). On this basis, we introduced mixed emotions and construal levels to further explore the impact mechanism of goal conflict on life satisfaction and tried to explain under what conditions and in what ways goal conflict affected wellbeing from the perspective of emotion and cognition.

### Goal Conflict and Life Satisfaction

Control theory points out that conflict within the same goal level will lead to pain, especially the conflicts between the high-level goals or the persistence of unresolved conflicts (Powers, 1974), and as a result, the goal conflict is associated with pain or low happiness. The negative predictive relationship between goal conflict and life satisfaction in this study verified the results of most studies in related fields (Emmons, 1986; Kelly et al., 2011; Gray et al., 2017). The latest theory of motivation and personality also holds that the individual behavioral inhibition system will be activated when incompatible behavior with similar strength occurs, and the function of this system is risk assessment, checking the source of the threat, as well as inhibiting ongoing behavior. The activation of the behavioral inhibition system may lead to an increase in negative evaluations of conflict stimuli, leading to rumination and worry (Corr and Krupić, 2017). The repressed goal progress is also often associated with increased psychological stress and decreased mental health (Boudreaux and Ozer, 2013; Sheldon et al., 2015); for example, studies have found that depressed groups report more conflict than healthy controls, and the association between goal conflict and psychological distress may be stronger in clinical populations (Feixas et al., 2014).

A noteworthy phenomenon is that the negative prediction of life satisfaction caused by goal conflict is not stable. Not all empirical studies have shown the negative association between goal conflict and happiness (Gorges et al., 2014; Gray et al., 2017). In the present study, there was a change in the relationship between goal conflict and life satisfaction. Without considering mixed emotions and construal level, there was a significant negative correlation between goal conflict and life satisfaction, and the former had a significant predictive effect on the latter. However, when mixed emotions and construal level were added as mediating variable and moderating variable respectively, the path coefficient was no longer significant (see Figure 2), and even became positive (see Table 3). The change in the path coefficient presenting an interesting result, it is not inevitable that goal conflict leads to a decrease in life satisfaction. Different levels, types, and durations of goal conflict have different effects on life satisfaction. Conflicts between low-level goals are less important in determining happiness than conflicts at other levels of the hierarchy (Kelly et al., 2015). Short-duration and conscious goal conflict may be less harmful. If individuals are aware that the goal they are pursuing is conflicting, they can resolve it by giving up a goal or by mobilizing more resources, but unconscious conflict is not easily resolved (Higginson et al., 2011). Therefore, the heterogeneity of goal conflict among subjects in the present study may be one of the reasons for the change in the prediction coefficient. In addition, the mediators and moderators related to individual emotional response and cognitive characteristics that we will discuss next are another important reason.

### Mediating Effect of Mixed Emotions

The results of the present study showed that mixed emotions play a mediating role between goal conflict and life satisfaction, and the impact of goal conflict on life satisfaction is achieved mainly through mixed emotions. This result was consistent with that of other scholars about the mediating effect of emotions (Kong et al., 2019; Lu et al., 2020). The balance between positive and negative emotions is crucial for life satisfaction judgment. Meanwhile, emotion is an important mediating factor for individual differences affecting life satisfaction (Kuppens et al., 2008; Zhu, 2015). The physiological model proposed by Salovey et al. (2000) holds that personality triggers physiological changes through various emotions, which in turn have an impact on health and wellbeing. Zeidner et al. (2012) also suggest that positive and negative emotions may play a mediating role in the relationship between emotional intelligence and life satisfaction.

However, the previous studies tend to regard positive and negative emotions as two separate parts and examine their roles, respectively (Zhu, 2015; Lu et al., 2020), which is difficult to suit the situation of goal conflict containing both positive information and negative information. It is more suitable for the present study to use mixed emotions as a mediating variable, and the results of this study have also proved this choice was appropriate. More than 90% of the effect of goal conflict on life satisfaction was achieved by mixed emotions. If we can change any of the mediating paths, we can significantly change the relationship between goal conflict and life satisfaction, and reduce the negative impact of goal conflict on happiness. For instance, if we could integrate conflicting goals at a higher level and increase the self-harmony degree of the goals (Gorges et al., 2014), then we could change the individual’s emotional experience to a certain extent, thereby improving life satisfaction. We can also improve individuals’ tolerance and coping ability to mixed emotional conflict and ambivalence (Hong and Lee, 2010; Mejía and Hooker, 2017), or change the mode of mixed emotion experience (Braniecka et al., 2014) to reduce the negative effects of mixed emotions on life. In addition, from the perspective of emotional regulation, choosing appropriate emotional regulation strategies, such as cognitive reappraisal or expression inhibition, to reduce the intensity of mixed emotions is also an alternative approach that individuals can try.

Mixed emotions are complex emotions in higher levels of the emotional structure (Watson and Stanton, 2017). The mediating role of mixed emotions between goal conflict and life satisfaction presented in this study reflected the important function of complex emotions between the individual difference and wellbeing, which is an expansion of previous studies on pure emotions. Compared to pure positive or negative emotions, mixed emotions are a better description of the complexity of life (Heavey et al., 2017; Burkitt et al., 2019), with better ecological validity.

### Moderating Effect of Construal Level

In the present study, it was found that the construal level had a significant moderating effect on the mediating path by which goal conflict influenced life satisfaction through mixed emotions. Individuals with a high construal level showed higher life satisfaction, especially when they were faced with medium or weak mixed emotions. This result was consistent with the relevant theoretical and empirical studies (Trope and Liberman, 2003; Hong and Lee, 2010; Gorges et al., 2014). The construal level theory states that a high construal level represents an overall generalization of events and high-level processing (Trope and Liberman, 2003), and is more likely to make that individuals have a long-term perspective and future-oriented thinking. A high construal level can effectively ease ambivalence and discomfort, and leads individuals to a more positive attitude toward goal conflict and mixed emotions (Hong and Lee, 2010; Mejía and Hooker, 2017), ultimately leading an individual to give a more positive assessment of life satisfaction. In addition, empirical research also shows that integration and higher-level values are the strategies that are often focused on the process of resolving the conflict of goals, and comprehensive solutions can help people promote multiple meaningful goals at the same time (Köpetz et al., 2011). In this case, the various aspects of self tend to be integrated into a relatively harmonious whole, with healthy psychological functions and happy experiences emerging.

The construal level can be presented either as a stable individual difference (Vallacher and Wegner, 1989) or as a state cognitive characteristic that can be changed (Liberman and Förster, 2009; Hong and Lee, 2010), which provides two different approaches for manipulating and changing the relationship between goal conflict, mixed emotions, and happiness by construal level. From a long-term and developmental perspective, the individuals can develop their own comprehensive and integrated habits of thinking, and reinforce their own cognitive tendency to process information at a high level, in order to improve the ability to deal with conflicts and mixed emotions. From the perspective of the short-term and situational adaptation, the individuals also can consciously keep themselves at the higher construal level when they are faced with conflicts and mixed emotions, processed the information in a holistic and general way to achieve the purpose of merging differences and mitigating ambivalence.

In addition, the present study also showed that the moderating effect of construal level was not obvious under the condition of high mixed emotions, and there was no significant difference in life satisfaction between individuals with high and low construal levels (see Figure 3). This may be due to a high level of mixed emotion which reflects the conflicts among high-level goals or internal goals. This type of conflict involves contradictions in self-worth and motivation, which is difficult to be integrated easily, so in this case, the relationship between goal conflict and wellbeing appears stable and strong negative contact (Kelly et al., 2015; Gray et al., 2017). The moderating effect of construal level has certain boundaries and limits, and its role between goal conflict and wellbeing should be viewed objectively.

### Limitations and Future Research Suggestions

The present study focused on the impact of goal conflict intensity on life satisfaction, without taking the other aspect of differences between goal conflicts into account. The goal conflict measured in the present study may be heterogeneous, which may result in the negative prediction of life satisfaction by goal conflict and is not robust enough. Goal conflicts with different levels, types, and durations have different effects on life satisfaction (Higginson et al., 2011; Kelly et al., 2015). In future research, it can be considered to separate the goal conflicts according to their different nature and compare the effects of different goal conflicts on wellbeing. This approach would be more conducive to grasp the different states of the relationship between goal conflict and wellbeing. In addition, this study is only preliminary correlational research, and future experimental studies are needed to verify the existing results.

Compared with pure positive or negative emotions, mixed emotions can better reflect the complexity of an individual’s life (Heavey et al., 2017; Burkitt et al., 2019) and better explain how complex situations affect happiness. The present study only focused on a complex situation of goal conflict, and the results can just make inferences in a limited range. In daily life, situations associated with mixed emotions are ubiquitous, such as work–family conflict, academic and environmental adaptation (Heavey et al., 2017), crisis management (Xiao et al., 2018), and major decision making (Mejía and Hooker, 2017). Future studies can verify and expand the current research by examining the influence of mixed emotions on wellbeing in different situations, so as to reach a more general conclusion.

The previous studies have shown that, in addition to the construal level, other factors can affect the individuals’ attitude and their response to conflicts and mixed emotions, such as dialectical thinking, Eastern and Western cultures, and age, which are closely related to wellbeing (Miyamoto and Ryff, 2011; Elliot et al., 2012; Liu et al., 2013). Future research can investigate the moderating effect of different individual characteristics and background factors on the relationship between goal conflict and wellbeing, so as to achieve a comprehensive understanding of the influencing mechanism between the two.

## Conclusion

Based on our analysis and discussion, the current study suggests that mixed emotions mediate the relationship between goal conflict and life satisfaction. Construal level moderated the second half of the mediation process, in which goal conflict affected life satisfaction through mixed emotions.

The present study provided a theoretical and empirical basis for correctly understanding and dealing with the relationship between goal conflict and happiness. According to the moderated mediating effect model established by us, goal conflict does not necessarily cause a decrease in life satisfaction, and some related cognitive and emotional variables will affect the relationship between the two. This conclusion provides an explanation for the inconsistent findings in the field. In addition, based on the above conclusions, we can propose to effectively intervene the negative impact of goal conflict on life satisfaction by reducing the intensity of emotional response or through emotional regulation. For example, when individuals are faced with mixed emotions caused by target conflict, they can use a higher construal level to regulate ambivalence and conflict sensitivity, so as to protect happiness.

## Data Availability Statement

The original contributions presented in the study are included in the article/supplementary material, further inquiries can be directed to the corresponding author.

## Ethics Statement

The studies involving human participants were reviewed andapproved by Capital Normal University. The patients/participants provided their written informed consent to participate in this study.

## Author Contributions

PF contributed to the conception and design of the study. ZZ and WS performed the statistical analysis, and WS wrote the first draft of the manuscript. PF and YJ revised it critically for important intellectual content. ZZ and LT collected the raw data and organized the database. All authors contributed to the article and approved the submitted version.

### Conflict of Interest

The authors declare that the research was conducted in the absence of any commercial or financial relationships that could be construed as a potential conflict of interest.

## Footnotes

^1^https://www.wjx.cn/

## References

[ref1] Austin J. T. Vancouver J. B. (1996). Goal constructs in psychology: structure, process, and content. Psychol. Bull. 120, 338–375. 10.1037/0033-2909.120.3.338

[ref2] Barford K. A. Koval P. Kuppens P. Smillie L. D. (2020). When good feelings turn mixed: affective dynamics and big five trait predictors of mixed emotions in daily life. Eur. J. Personal. 34, 393–411. 10.1002/per.2264

[ref3] Bee C. C. Madrigal R. (2013). Consumer uncertainty: the influence of anticipatory emotions on ambivalence, attitudes, and intentions. J. Consum. Behav. 12, 370–381. 10.1002/cb.1435

[ref4] Berrios R. Totterdell P. Kellet S. (2015a). Investigating goal conflict as a source of mixed emotions. Cognit. Emot. 29, 755–763. 10.1080/02699931.2014.939948, PMID: 25040183

[ref5] Berrios R. Totterdell P. Kellett S. (2015b). Eliciting mixed emotions: a meta-analysis comparing models, types, and measures. Front. Psychol. 6:428. 10.3389/fpsyg.2015.00428 25926805 PMC4397957

[ref6] Berrios R. Totterdell P. Kellett S. (2017). Individual differences in mixed emotions moderate the negative consequences of goal conflict on life purpose. Personal. Individ. Differ. 110, 18–22. 10.1016/j.paid.2017.01.013

[ref7] Berrios R. Totterdell P. Kellett S. (2018). When feeling mixed can be meaningful: the relation between mixed emotions and eudaimonic well-being. J. Happiness Stud. 19, 841–861. 10.1007/s10902-017-9849-y

[ref8] Bodner E. Bergman Y. S. Cohen-Fridel S. (2014). Do attachment styles affect the presence and search for meaning in life? J. Happiness Stud. 15, 1041–1059. 10.1007/s10902-013-9462-7

[ref9] Boudreaux M. J. Ozer D. J. (2013). Goal conflict, goal striving, and psychological wellbeing. Motiv. Emot. 37, 433–443. 10.1007/s11031-012-9333-2

[ref10] Braniecka A. Trzebińska E. Dowgiert A. Wytykowska A. (2014). Mixed emotions and coping: the benefits of secondary emotions. PLoS One 9, 1–13. 10.1371/journal.pone.0103940 PMC4118988 25084461

[ref11] Burkitt E. Watling D. Cocks F. (2019). Mixed emotion experiences for self or another person in adolescence. J. Adolesc. 75, 63–72. 10.1016/j.adolescence.2019.07.004, PMID: 31349096

[ref12] Cacioppo J. T. Berntson G. G. (1994). Relationship between attitudes and evaluative space: a critical review, with emphasis on the separability of positive and negative substrates. Psychol. Bull. 115, 401–423. 10.1037/0033-2909.115.3.401

[ref13] Carrera P. Fernández I. Muñoz D. Caballero A. (2020). Using abstractness to confront challenges: how the abstract construal level increases people's willingness to perform desirable but demanding actions. J. Exp. Psych. Appl. 26, 339–349. 10.1037/xap0000244, PMID: 31535885

[ref14] Corr P. J. Krupić D. (2017). Chapter Two -Motivating personality: approach, avoidance, and their conflict. Advances in Motivation Science 4, 39–90. 10.1016/bs.adms.2017.02.003

[ref15] Covington M. V. (2000). Goal theory, motivation, and school achievement: an integrative review. Annu. Rev. Psychol. 51, 171–200. 10.1146/annurev.psych.51.1.171, PMID: 10751969

[ref16] Deci E. L. Ryan R. M. (2008). Self-determination theory: a macrotheory of human motivation, development, and health. Can. Psychol. 49, 182–185. 10.1037/a0012801

[ref17] DeYoung C. G. (2015). Cybernetic big five theory. J. Res. Pers. 56, 33–58. 10.1016/j.jrp.2014.07.004

[ref18] Diener E. D. Emmons R. A. Larsen R. J. Griffin S. (1985). The satisfaction with life scale. J. Pers. Assess. 49, 71–75. 10.1207/s15327752jpa4901_13, PMID: 16367493

[ref19] Elliot A. J. Sedikides C. Murayama K. Tanaka A. Thrash T. M. Mapes R. R. (2012). Cross-cultural generality and specificity in self-regulation: avoidance personal goals and multiple aspects of well-being in the United States and Japan. Emotion 12, 1031–1040. 10.1037/a0027456, PMID: 22506500

[ref20] Emmons R. A. (1986). Personal strivings: an approach to personality and subjective well-being. J. Pers. Soc. Psychol. 51, 1058–1068. 10.1037/0022-3514.51.5.1058

[ref21] Emmons R. A. King L. A. (1988). Conflict among personal strivings: immediate and long-term implications for psychological and physical well-being. J. Pers. Soc. Psychol. 54, 1040–1048. 10.1037/0022-3514.54.6.1040, PMID: 3397863

[ref22] Feixas G. Montesano A. Erazo-Caicedo M. I. Compañ V. Pucurull O. (2014). Implicative dilemmas and symptom severity in depression: a preliminary and content analysis study. J. Constr. Psychol. 27, 31–40. 10.1080/10720537.2014.850369

[ref23] Fernández I. Caballero A. Muñoz D. Aguilar P. Carrera P. (2018). Abstract construal level and its link to self-control and to cross-situational consistency in self-concept: predicting health-risk behavioral intentions. Span. J. Psychol. 21, 1–8. 10.1017/sjp.2018.43, PMID: 30355389

[ref24] Förster J. Friedman R. S. Liberman N. (2004). Temporal construal effects on abstract and concrete thinking: consequences for insight and creative cognition. J. Pers. Soc. Psychol. 87, 177–189. 10.1037/0022-3514.87.2.177, PMID: 15301626

[ref25] Freitas A. L. Clark S. L. Kim J. Y. Levy S. R. (2009). Action-construal levels and perceived conflict among ongoing goals: implications for positive affect. J. Res. Pers. 43, 938–941. 10.1016/j.jrp.2009.05.006

[ref26] Freund A. M. Hennecke M. (2015). On means and ends: the role of goal focus in successful goal pursuit. Curr. Dir. Psychol. Sci. 24, 149–153. 10.1177/0963721414559774

[ref27] Fujita K. Trope Y. Liberman N. Levin-Sagi M. (2006). Construal levels and self-control. J. Pers. Soc. Psychol. 90, 351–367. 10.1037/0022-3514.90.3.351, PMID: 16594824 PMC3153425

[ref28] Gorges J. Esdar W. Wild E. (2014). Linking goal self-concordance and affective reactions to goal conflict. Motiv. Emot. 38, 475–484. 10.1007/s11031-014-9392-7

[ref30] Gray J. S. Ozer D. J. Rosenthal R. (2017). Goal conflict and psychological well-being: a meta-analysis. J. Res. Pers. 66, 27–37. 10.1016/j.jrp.2016.12.003

[ref31] Hayes A. F. (2015). An index and test of linear moderated mediation. Multivar. Behav. Res. 50, 1–22. 10.1080/00273171.2014.962683, PMID: 26609740

[ref32] Heavey C. L. Lefforge N. L. Lapping-Carr L. Hurlburt R. T. (2017). Mixed emotions: toward a phenomenology of blended and multiple feelings. Emot. Rev. 9, 105–110. 10.1177/1754073916639661

[ref33] Hershfield H. E. Scheibe S. Sims T. L. Carstensen L. L. (2013). When feeling bad can be good: mixed emotions benefit physical health across adulthood. Soc. Psychol. Personal. Sci. 4, 54–61. 10.1177/1948550612444616, PMID: 24032072 PMC3768126

[ref34] Higginson S. Mansell W. Wood A. M. (2011). An integrative mechanistic account of psychological distress, therapeutic change and recovery: the perceptual control theory approach. Clin. Psychol. Rev. 31, 249–259. 10.1016/j.cpr.2010.01.005, PMID: 20171771

[ref35] Hong J. Lee A. Y. (2010). Feeling mixed but not torn: the moderating role of construal level in mixed emotions appeals. J. Consum. Res. 37, 456–472. 10.1086/653492

[ref36] Hooker K. (2015). Towards a new synthesis for development in adulthood. Res. Hum. Dev. 12, 229–236. 10.1080/15427609.2015.1068036

[ref37] Hull C. L. (1938). The goal-gradient hypothesis applied to some field-force'problems in the behavior of young children. Psychol. Rev. 45, 271–299. 10.1037/h0053885

[ref38] Kelly R. E. Mansell W. Wood A. M. (2011). Goal conflict and ambivalence interact to predict depression. Personal. Individ. Differ. 50, 531–534. 10.1016/j.paid.2010.11.018

[ref39] Kelly R. E. Mansell W. Wood A. M. (2015). Goal conflict and well-being: a review and hierarchical model of goal conflict, ambivalence, self-discrepancy and self-concordance. Personal. Individ. Differ. 85, 212–229. 10.1016/j.paid.2015.05.011

[ref40] King L. A. Richards J. H. Stemmerich E. (1998). Daily goals, life goals, and worst fears: means, ends, and subjective well-being. J. Pers. 66, 713–744. 10.1111/1467-6494.00030, PMID: 9802231

[ref41] Kong F. Gong X. Sajjad S. Yang K. Zhao J. (2019). How is emotional intelligence linked to life satisfaction? The mediating role of social support, positive affect and negative affect. J. Happiness Stud. 20, 2733–2745. 10.1007/s10902-018-00069-4

[ref42] Köpetz C. Faber T. Fishbach A. Kruglanski A. W. (2011). The multifinality constraints effect: how goal multiplicity narrows the means set to a focal end. J. Pers. Soc. Psychol. 100, 810–826. 10.1037/a0022980, PMID: 21381854

[ref43] Kuppens P. Realo A. Diener E. (2008). The role of positive and negative emotions in life satisfaction judgment across nations. J. Pers. Soc. Psychol. 95, 66–75. 10.1037/0022-3514.95.1.66, PMID: 18605852

[ref44] Larsen J. T. (2017). Introduction to the special section on mixed emotions. Emot. Rev. 9, 97–98. 10.1177/1754073916672523

[ref45] Larsen J. T. McGraw A. P. Cacioppo J. T. (2001). Can people feel happy and sad at the same time? J. Pers. Soc. Psychol. 81, 684–696. 10.1037/0022-3514.81.4.684, PMID: 11642354

[ref46] Liberman N. Förster J. (2009). Distancing from experienced self: how global-versus-local perception affects estimation of psychological distance. J. Pers. Soc. Psychol. 97, 203–216. 10.1037/a0015671, PMID: 19634971

[ref47] Liu S. Peng K. Liu G. Fang P. Lin Z. Li D. (2013). Bian zheng qing xu: yan jiu fang fa yu zhang wang [Dialectical emotion: research methods and prospects]. Xin Li Xue Tan Xin 33, 7–14.

[ref48] Locke E. A. Latham G. P. (2002). Building a practically useful theory of goal setting and task motivation: a 35-year odyssey. Am. Psychol. 57, 705–717. 10.1037/0003-066X.57.9.705 12237980

[ref49] Lu C. Jiang Y. Zhao X. Fang P. (2020). Will helping others also benefit you? Chinese adolescents’ altruistic personality traits and life satisfaction. J. Happiness Stud. 21, 1407–1425. 10.1007/s10902-019-00134-6

[ref50] Maslow A. H. (1943). Conflict, frustration, and the theory of threat. J. Abnorm. Soc. Psychol. 38, 81–86. 10.1037/h0054634

[ref51] McNaughton N. Gray J. A. (2000). Anxiolytic action on the behavioural inhibition system implies multiple types of arousal contribute to anxiety. J. Affect. Disord. 61, 161–176. 10.1016/S0165-0327(00)00344-X, PMID: 11163419

[ref52] Mejía S. T. Hooker K. (2017). Mixed emotions within the context of goal pursuit. Curr. Opin. Behav. Sci. 15, 46–50. 10.1016/j.cobeha.2017.05.015, PMID: 29201977 PMC5703421

[ref53] Miyamoto Y. Ryff C. D. (2011). Cultural differences in the dialectical and non-dialectical emotional styles and their implications for health. Cognit. Emot. 25, 22–39. 10.1080/02699931003612114, PMID: 21432654 PMC3269302

[ref54] Oceja L. Carrera P. (2009). Beyond a single pattern of mixed emotional experience. Eur. J. Psychol. Assess. 25, 58–67. 10.1027/1015-5759.25.1.58

[ref55] Pavot W. Diener E. (1993). Review of the satisfaction with life scale. Psychol. Assess. 5, 164–172. 10.1037/1040-3590.5.2.164

[ref56] Penz E. Hogg M. K. (2011). The role of mixed emotions in consumer behaviour: investigating ambivalence in consumers' experiences of approach – avoidance conflicts in online and offline settings. Eur. J. Mark. 45, 104–132. 10.1108/03090561111095612

[ref57] Perring C. Oatley K. Smith J. (1988). Psychiatric symptoms and conflict among personal plans. Br. J. Med. Psychol. 61, 167–177. 10.1111/j.2044-8341.1988.tb02776.x 3401425

[ref58] Podsakoff P. M. MacKenzie S. B. Lee J. Y. Podsakoff N. P. (2003). Common method biases in behavioral research: a critical review of the literature and recommended remedies. J. Appl. Psychol. 88, 879–903. 10.1037/0021-9010.88.5.879 14516251

[ref100] Powers W. T. (1974). Behaving man. (book reviews: behavior. the control of perception). Science 184, 455–457. 10.1126/science.184.4135.455, PMID: 18808239

[ref101] Raykov T. Dimitrov D. M. Asparouhov T., (2010). Evaluation of scale reliability with binary measures using latent variable modeling. Struct. Equ. Modeling, 17, 265–279. 10.1080/10705511003659417, PMID: 18808239

[ref60] Riediger M. Freund A. M. (2008). Me against myself: motivational conflicts and emotional development in adulthood. Psychol. Aging 23, 479–494. 10.1037/a0013302, PMID: 18808239

[ref61] Romero E. Villar P. Luengo M. Á. Gómez-Fraguela J. A. (2009). Traits, personal strivings and well-being. J. Res. Pers. 43, 535–546. 10.1016/j.jrp.2009.03.006

[ref62] Salovey P. Rothman A. J. Detweiler J. B. Steward W. T. (2000). Emotional states and physical health. Am. Psychol. 55, 110–121. 10.1037/0003-066X.55.1.110 11392855

[ref63] Sheldon K. M. Jose P. E. Kashdan T. B. Jarden A. (2015). Personality, effective goal-striving, and enhanced well-being: comparing 10 candidate personality strengths. Personal. Soc. Psychol. Bull. 41, 575–585. 10.1177/0146167215573211, PMID: 25713170

[ref64] Shuman V. Sander D. Scherer K. R. (2013). Levels of valence. Front. Psychol. 4:261. 10.3389/fpsyg.2013.00261, PMID: 23717292 PMC3651968

[ref65] Sun W. Jiang Y. Fang P. (2021). Hun he qing xu neng cu jin jian kang ma? [Can mixing emotions promote mental health]. Xin Li Ke Xue 44, 230–236. 10.16719/j.cnki.1671-6981.20210132

[ref66] Trope Y. Liberman N. (2003). Temporal construal. Psychol. Rev. 110, 403–421. 10.1037/0033-295X.110.3.403, PMID: 12885109

[ref67] Unsworth K. Yeo G. Beck J. (2014). Multiple goals: a review and derivation of general principles. J. Organ. Behav. 35, 1064–1078. 10.1002/job.1963

[ref68] Vallacher R. R. Wegner D. M. (1989). Levels of personal agency: individual variation in action identification. J. Pers. Soc. Psychol. 57, 660–671. 10.1037/0022-3514.57.4.660 2926623

[ref69] Watson D. Stanton K. (2017). Emotion blends and mixed emotions in the hierarchical structure of affect. Emot. Rev. 9, 99–104. 10.1177/1754073916639659

[ref70] West R. L. Ebner N. C. Hastings E. C. (2013). “Linking goals and aging: experimental and lifespan approaches” in New Developments in Goal Setting and Task Performance. eds. Locke E. A. Latham G. P. (New York, NY: Routledge), 463–483.

[ref102] Wong N. Y. Bagozzi R. P. (2005). Emotional intensity as a function of psychological distance and cultural orientation. J. Bus. Res. 58, 533–542. 10.1016/S0148-2963(03)00144-9

[ref71] Xiao Y. Hudders L. Claeys A. S. Cauberghe V. (2018). The impact of expressing mixed valence emotions in organizational crisis communication on consumer’s negative word-of-mouth intention. Public Relat. Rev. 44, 794–806. 10.1016/j.pubrev.2018.10.007

[ref72] Zeidner M. Matthews G. Roberts R. D. (2012). The emotional intelligence, health, and well-being nexus: what have we learned and what have we missed? Appl. Psychol. Health Well Being 4, 1–30. 10.1111/j.1758-0854.2011.01062.x, PMID: 26286968

[ref73] Zhu H. (2015). Social support and affect balance mediate the association between forgiveness and life satisfaction. Soc. Indic. Res. 124, 671–681. 10.1007/s11205-014-0790-8

